# Correction: Sekar et al. Concussion/Mild Traumatic Brain Injury (TBI) Induces Brain Insulin Resistance: A Positron Emission Tomography (PET) Scanning Study. *Int. J. Mol. Sci.* 2021, *22*, 9005

**DOI:** 10.3390/ijms27135650

**Published:** 2026-06-23

**Authors:** Sathiya Sekar, Raja Solomon Viswas, Hajar Miranzadeh Mahabadi, Elahe Alizadeh, Humphrey Fonge, Changiz Taghibiglou

**Affiliations:** 1 Department of Anatomy, Physiology and Pharmacology, College of Medicine, University of Saskatchewan, 107 Wiggins Road, Saskatoon, SK S7N 5E5, Canada; sathiya.sekar@usask.ca (S.S.); h.miranzadeh@usask.ca (H.M.M.); 2 Department of Medical Imaging, College of Medicine, University of Saskatchewan, Saskatoon, SK S7N 0W8, Canada; rav137@mail.usask.ca (R.S.V.); elahe.alizadeh@usask.ca (E.A.); 3 Department of Medical Imaging, Royal University Hospital (RUH), Saskatoon, SK S7N 0W8, Canada

In the original publication [1], there was a mistake in Figure 5D,E. The new image Figure 5D contains an updated (correct) scan of our KO blot. Regarding Figure 5E, we could not locate the original blots for two of the test conditions; we, therefore, deleted panel 5E and revised the figure legend. The corrected Figure 5 and the legend appear below.

**Figure 5:**
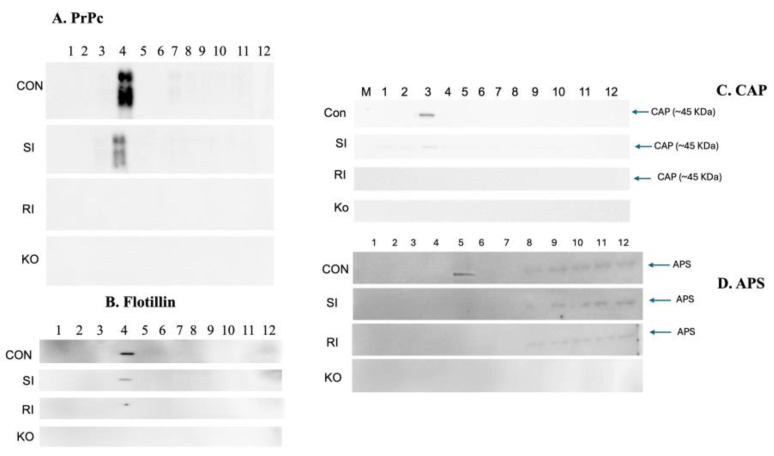
Determination of PrPc and CAP/Cbl signaling protein levels in the lipid raft region of TBI mouse brains. Levels of PrPc (**A**), flotillin (**B**), CAP (**C**), and APS (**D**) in lipid raft regions of control, single and repeated TBI-induced mouse, and PrPc-null mouse brains were measured using lipid raft isolation followed by Western blotting technique (*n* = 3). CON—control; SI—single induction; RI—repeated induction; KO—PrPc-null mouse brains.

Accordingly, a correction has been made to Section 2.4. “*Alterations of PrPc, Flotillin, CAP, and APS Proteins in Lipid Raft Region of TBI Mouse Brains Following Single and Repeated Inductions*”

For further behavioral assessment, the mice were sacrificed and their brains excised out. The lipid raft regions from the brains were isolated by the sucrose gradient centrifugation method. Twelve fractions were separated in each sample and blotted for PrPc, flotillin, CAP, and APS proteins. The curdy white precipitate was collected as the 4th fraction that was considered to be the lipid raft fraction. The results obtained from the present study showed that these proteins were markedly decreased in single induction mice compared with control mouse brains, while no expression was observed in repeated induction mice similar to PrPc-null mouse brains (Figure 5).

The authors state that the scientific conclusions are unaffected. This correction was approved by the Academic Editor. The original publication has also been updated.
